# An Accidental Revolution: The ILO and the Opening Up of International Law

**DOI:** 10.1007/978-3-030-55400-2_6

**Published:** 2020-10-17

**Authors:** Jan Klabbers

**Affiliations:** 1 Office of the President of the Republic of Finland, Helsinki, Finland; 2 Faculty of Law, University of Helsinki, Helsinki, Finland; University of Helsinki, Helsinki, Finland

## Abstract

This article discusses the pioneering role of the ILO not in terms of its contribution to labour law, but in terms of its epistemic relevance: it was the first international organization which cut through the classic borderline between national law and international law. In order to do so, the article sketches pre-ILO legal doctrine, and discusses the creation and particular structure of the ILO at some length: why even create an organization to address labour issues, instead of concluding a convention? This is followed by outlining just how relevant the role of the ILO has been.

## Introduction

Contrary to popular opinion, the role of law, both in domestic societies and in international affairs, is not first and foremost about constraining action. Law is not about telling people how to behave, and inflicting punishment when they behave differently—not solely, at any rate. To think this, as many do, is to view criminal law as the template for law generally. Instead, much of the law, both in domestic societies and in international affairs, follows a different template, and is about facilitating action.

What is more, in addition to (or in the process of) facilitating action, law also helps to structure the way we think about things. We cannot begin to think of the state without invoking the criteria for statehood; we cannot seriously discuss agreement without bearing legal concepts of treaty or contract in mind; and we cannot characterize the military presence of state A in state B without some term from the legal vocabulary, and it matters a great deal which exact term we employ, for calling something an “invasion” or an “attack” evokes different associations than labelling the same act an “intervention”.^1^ That is not to say our conversations should stop at those legal concepts, for sometimes doing so might lapse into awkwardness or worse, as when a court proves unable to think of genocide in terms other than those of the 1948 Genocide Convention and thus suggests that an earlier genocide was probably not “really” a “genocide”.^2^ That said, though, rules, norms and decisions^3^ set the tone for any social conversation. Rules, norms and decisions also tend to have distributive effects. Any authoritative decision will allocate something of value, whether financial or social. A decision by a country club to admit someone as a member will change that individual’s relative standing in the community, and a decision by the UN Educational, Scientific and Cultural Organization (UNESCO) to admit Palestine as a member adds legitimacy to Palestine’s position in global politics. A decision by a pizza parlour to change its opening hours or update its menu will convenience some and inconvenience others. Enacting a rule that inaugurs driving on the right side of the road will disadvantage some car manufacturers, even if one might hold that the rule is a textbook example of a coordination rule. And a decision by the World Health Organization (WHO) to declare a pandemic will come to affect the producers of vaccines, may send shockwaves through the tourism industry, and may even inaugurate a full-blown economic crisis, as the 2020 Covid-19 crisis vividly illustrates.^4^

Given that rules, norms and decisions invariably have distributive effects, they are typically employed as weapons and arms in struggles for power and hegemony between people (states, companies, organizations, individuals) with diverging political agendas. Those weapons may have long fuses, and their effects may only manifest themselves over time, but this makes them only more effective, for the most effective form of power is the power to influence how people think about things.^5^ No lesser authority than John Maynard Keynes was well aware of this, explicitly dedicating his analysis of the Versailles settlement to influencing the minds of future generations of policy makers.^6^

With this in mind, the creation of the ILO can be seen as a crucial step in the development of public international law, and its singular relevance resides in having sensitized international law to addressing the situation of individuals, whether as employers or as workers. The relevance of the ILO is not just that it helped create and enforce labour rights, although it did and does that too. But part of its relevance also resides in something else, on a deeper level so to speak; this has little to do with labour rights per se, but rather more with opening up international law, with making visible that international law is not just about inter-state relations. The ILO is probably the first international organization—the first manifestation of international law—to take individuals and companies seriously, thus paving the way for the involvement of international law in more recent years with individuals, something we now almost take for granted. Human rights involve the individual, as do international criminal law, EU law, the law on investment protection, et cetera. It is impossible to prove (and silly even to try) that none of this would have happened without the ILO. But what can be demonstrated is that the ILO marked a significant step in the creation of the individual as an entity of relevance to international law.^7^

In what follows, I will substantiate that particular claim, demonstrating first that the international legal vocabulary prior to the ILO’s creation did not facilitate thinking about individual rights under international law, in thrall as it was to the idea that international law only operated between states and would only affect states, in their capacity as states. Thereafter, I will discuss the creation of the ILO, indicating just how creating the ILO marked a seismic shift. This is followed by a discussion as to how and why the international legal vocabulary—the landscape—changed with the establishment of the ILO.

## Dualism and Its Discontents

Traditionally, international law was always nominally concerned with relations between states. International law, in a collated textbook definition from the late nineteenth century, was the law made by states, to regulate relations between states, and for the benefit of those states. Oppenheim, e.g., in the second edition of his classic treatise published in 1912, defined international law as “the body of customary and convention rules which are considered legally binding by civilized States in their intercourse with each other”.^8^ And he adds that international offices are created to give effect to treaties establishing unions between states.^9^ There was not a hint of a suggestion here that international law, or the work of those international offices, might come to affect others than states. States enjoyed, one might say, considerable epistemic priority. Other actors never even entered the picture in any serious manner, except as religious or historical exceptions.^10^ After all, so the logic went, only states can go to war. Only states can conclude treaties. Only states can proclaim territorial waters.

The epistemic priority of the state also extended to international organizations. These were always derivative creatures, deriving their existence and powers from the states that founded them.^11^ What is more, international organizations were not supposed to have any outward-radiating effect. If the early international organizations were not endowed with international legal personality, it was because no one considered such personality necessary, for the good reason that organizations were not supposed to interact directly with anyone other than their member states—not with third states, not with other international organizations, and not with the citizens of their member states either. And for much the same reason, they had no treaty-making powers to speak of. Each organization was supposed to be a universe onto itself (*res inter alios acta*), with the only relationships envisaged being those between the organization and its member states, but never with the outside world.^12^ This still applied, in 1945, to the UN, set up as an entity of universal scope both substantively and in terms of geographical reach, but with few treaty-making powers or even provisions recognizing that there existed a world outside the organization (military agreements were envisaged to regulate troop contributions, and some coordination with other organizations was planned, but not much more) and no explicit grant of international legal personality. The latter only came about after the intervention of the International Court of Justice, in 1949.^13^

And when international law even deigned to think about individuals, it was only in relation to the state, only as state representatives. This applied formally with respect to protection of diplomats or the conclusion of treaties. It applied more artificially with the protection of property abroad: injuring the individual was seen as injuring the state, and entitling the state (though not the individual) to take action.^14^ Not everyone was convinced. Philip Jessup could write in the late 1940s that if injury to the state was the true basis of responsibility for injury to aliens, then “the measure of damages to be paid for an injury would vary with the importance of the role played by the injured individual in the life of the state of which he is a citizen.”^15^

All this suggested that international law and domestic law would never need to be in touch with one another: international law stayed on the inter-state level, and the rest was the concern of domestic law.^16^ The logic of thinking of international and domestic law as separate systems made some sense, on the surface level—otherwise it could not have survived for very long. It did however rest on one condition: it only made sense as long as no one asked why states would go to war and who would be affected by war; or why states concluded treaties or proclaim territorial waters, and who would be affected. Once those questions are asked, the idea of there being purely inter-state activities which form the natural realm of international law, quickly turns into a conceit.

But founded on the logic that international affairs are by definition merely inter-state affairs,^17^ no situation could possibly bring the individual into contact with international law, as indeed Triepel observed and further theorized in 1899.^18^ The universes of domestic law and international law were considered to be hermetically sealed off. Empirically, Triepel noted, domestic law deals with relations involving individuals, and international law is limited to regulating relations between states. On the rare occasions that a treaty would aim to do something for individuals, it would have to be transformed into domestic law. This idea came to be known as dualism, and is still maintained (albeit often in somewhat softened form) in many states. The gist is that domestic legal orders can only work on the basis of instruments recognized as legally valid within those domestic orders, typically Acts of Parliament, Governmental Decrees, and the like. As a result, other instruments, regardless of their provenance (but typically referring to international legal instruments) must be transformed into acts recognized as legally valid by and within the legal order concerned; a treaty must be transformed into an Act of Parliament or Governmental Decree in order to be recognized as valid within that legal order, and in order to create rights or obligations for individuals within that legal order.

This was never a fully accurate or convincing picture, but it worked until the 1920s, and generated an understandable popularity. It entailed that domestic parliaments, which had fought hard and long to acquire a say over domestic legislation, could not be outflanked or overruled by governments entering into international commitments. Over international commitments, after all, typically those same parliaments had no influence. If dualism thus respected concerns about local democracy (at least nominally), a side-effect was the re-affirmation of the role of the state and a re-affirmation of the strict separation between international and domestic law.

Triepel himself pointed out that his theory was empirically-based; it was built on the finding that there actually were no contacts between the international and the domestic legal order. These things are always in the eye of the beholder (in that few matters in law really have an empirical correspondent independently from the particular theory in which empirical observations play a role, and tend to be much more dependent on hermeneutics^19^), but Triepel made a forceful case. He did note, however, that the minorities treaties concluded in connection with the re-drawing of Europe’s map at Versailles could come to affect individuals.^20^ But, he wrote in 1923, that moment had not yet arrived. International law still dealt only with states.

Naturally, this strict separation between the domestic and international spheres also affected the creation of international organization, including the very early river commissions addressing issues of navigation, safety, and security. The idea behind the river commissions was to establish common rules for navigation, and this was done by ordering the states to legislate—thus keeping the separate spheres intact. The Final Act of the Congress of Vienna 1815, e.g., proves illustrative. Article 108 provides that states set up common regimes for navigation, while Article 110 made clear that “uniformity” was key. On the Rhine, the Neckar, and other rivers, the exact same rules should apply with respect to all states concerned, both relating to navigation and in terms of policing. Article 111 further underlined the need for harmonization: “Les droit sur la navigation seront fixé d’une maniére uniforme, invariable, et […] indépendente de la qualité différente des marchandises […]”.

Note the way Article 111 was written: what was needed here was for the river commissions to set standards, and then for the riparian states to turn these into law—no one had given any thought to allowing river commission to set those standards directly. Instead, the instruction of Article 111 was directed at states: states would have to set in uniform manner the navigation rights.

This pattern continued throughout the nineteenth and early twentieth century. The International Telegraphic Union (ITU), the Universal Postal Union (UPU), International Bureau of Weights and Measures, the Union of International Transport by Rail, the International Sugar Union, the International Institute for Agriculture: all late-nineteenth century and early twentieth century creations were thought of as creations of states, affecting those very states (the member states) in their very capacity as states. And in the case where they were not thought of in state-centric terms, as creatures of states, then they were not considered part of international law. The Red Cross (created in 1863 by Henri Dunant and Gustave Moynier^21^) is a prime example; another is the Institut de Droit International, set up in Ghent in 1873.^22^

Still, every now and then a minor crack became visible. The US, e.g., was reluctant to join the ITU,^23^ mostly because the telegraph networks in the US were in private hands, while in other member states they were usually under public control. This seemed to signify, however dimly, a realization that the work of the ITU might affect network operators. It was also said of the Union of International Transport by Rail that its dispute settlement procedures made no distinction between governmental and nongovernmental railway administration, again suggesting a dim realization that the Union’s work may affect entities within the state, and not just those states themselves.^24^

Indeed, the strict separation between the international and domestic spheres was never very realistic. It seems fairly obvious that the setting of postal rates by UPU not only affects Denmark and Japan and Nigeria, but also affects individuals and businesses as senders of letters and packages, and it seems fairly obvious that prices set by the sugar union affect the market price for sugar and therewith immediately affect consumers and producers.^25^ But while there was inevitably an indirect effect on individuals, it was always mediated by the state—and indeed, international law did not have any other mechanisms at its disposal. Edwin Borchard, writing in 1940, summarized the dualist position, noting that “dualists will admit that many of the rules of treaty and international law are devised for and accrue to the benefit of individuals, they nevertheless insist that only States may become spokesmen for these rules and advantages.”^26^

The thought that international law could have direct effect on individuals was, so to speak, not yet thought, and would only first be thought by the Permanent Court of International Justice (PCIJ) in the late 1920s.^27^ And even then, the PCIJ, when developing its position on direct effect, did so with considerable ambivalence: whether or not a provision of a treaty would be directly effective would depend on the intentions of the drafters of that provision, and those drafters were, invariably, states. This was, in other words, not quite the empirical position Triepel had in mind. Or rather, more accurately perhaps, the empirical evidence could be manipulated by states: a provision where international and domestic law would be in contact could still be said not to be directly effective if there would be an indication that parties wished to preclude direct effect.^28^

## Establishing the ILO

But in 1919, when the ILO was created in Versailles, this was still something for the future.

Versailles saw the creation of the League of Nations, the clearly still highly state-centric creature to guarantee collective security.^29^ But Versailles also saw the creation of the ILO. But why the ILO, and why not an international organization for, say, global health? Or for maritime affairs or arms control? Why even create a second organization, in addition to the League, and why not simply a convention to treat workers decently? In other words, what was the problem to which this international organization, the ILO, was expected to be the solution?

The obvious answer—or the beginning of an answer—is that the ILO marks a response to the October revolution of 1917, and reading contemporary papers and books, there is a clear sense of urgency on this point. Part of the idea behind the ILO was to set it up as an answer to the red threat, to communism. As David Morse, long-time Director-General of the ILO, much later put it in admirably bureaucratic and anodyne style, “there was general recognition that the ferment and instability which characterized the world of labor and industry in 1918 and 1919, particularly in Europe, called for immediate and constructive action.”^30^ The idea was to make the working man happy, or rather, to make sure he would not be so unhappy that he would turn to communism. In practice, this entailed decent working conditions: it is surely no coincidence that the first ILO conventions deal with working hours, unemployment, maternity protection, and night work. The first recommendations addressed similar matters and tried to protect against dangerous materials, aiming to protect works working with anthrax, lead, white phosphorus.^31^

But still, a set of intellectual problems emerged. The drafters realized all too well that economic circumstances differ from country to country, from state to state. Thus, there is a quasi-natural competitive obstacle that needs to be overcome. What made things more difficult still was the realization that protecting labour comes at the expense of capital, and that the costs and benefits might not be evenly distributed. Some industries would be harder hit than others; for some industries, protecting workers would come at bigger costs than for other industries, not because those others would have been doing so earlier, but because they would be less dependent on night work, or would be less involved with dangerous materials. A third problem that emerged revolved around colonialism: some of the bigger states benefitted from cheap labour being available in their colonies. In fact, as one of the founding fathers, Britain’s George Barnes, openly confessed in relation to the imperial issue: “To be quite candid, our motives were not altogether humanitarian.”^32^

Instead, while the communist threat was perceived as very real, it had to be met in such a way as not to distort global competition. The same George Barnes notes, in his work on the ILO written a few years after its creation, that the “need had arisen for levelling out industrial competition between the nations by raising the conditions of labour in the lower-paid countries”,^33^ and diagnosed the problem as being related to mass manufacturing by “cheap Eastern labour”.^34^

This proved quite a riddle. Capitalism requires competition, after all, and one of the more obvious arenas for industrial competition is in the sphere of labour, both by keeping wages low and not spending much on decent working conditions. Yet allowing for the race to the bottom to occur was thought to play in the hands of communism, putting the capitalist world economy at risk. It seemed a veritable catch-22: either allow for unhampered competition and invite communism to take over, or limit competition as far as labour issues are concerned and in that way implicitly accommodate communism as well. Clearly, this left a delicate balancing act: infusing just enough worker protection into the system so as to save the system: too much would make the capitalist economy collapse, and too little would have pretty much the same result. The logic was well-put by a contemporary observer, Leonard Woolf: “If it is in the interest of every State to regulate the conditions of employment within its territory, but it is prevented from doing so unless all the other States do likewise”, so Woolf wrote, “then clearly the solution ought to be found in unification of the Labour laws of the different countries through international agreements.”^35^

One possible way—hypothetically at any rate—to solve the problem was to leave it entirely to the market, and open the borders for unmitigated migration. If the capitalist logic would work, after all, then people would move to the place where there would be work and a decent wage. This, however, was never realistic. As John Hobson observed at the time, Asia may be a “rich reservoir” of labour, but “the difficulty of procuring the general assent of civilized nations to “an open door” for Asiatic labour would, of course, be insuperable”^36^; Hobson’s casual use of the term “of course” spoke volumes.

One thing that became reasonably clear was that a single convention on worker’s rights was unlikely to do the trick. What was needed instead was a careful and continued balancing of the interests of workers, capital and states, and this, in turned, required permanent management, not a one-off arrangement in the form of a treaty; for a single treaty could never be comprehensive enough to cover all industries, cover all kinds of situations that might arise, and accommodate all conflicting interests.^37^ And the balancing act turned out to be quite successful. As historian Emily Rosenberg concludes, generally speaking “the organization supported a liberal capitalist system operating through cooperating national states […] and opposed an alternative transnational labor movement that was being promoted through the Soviet Union’s Third International.”^38^

The ILO’s success in performing the balancing act of navigating between unfettered labour competition possibly leading to communism, and adopting communism *tout court*, it can be said with considerable hindsight, was due to the combination of organizational form and tripartite structure—even if the precise limits of this organizational form remained subject to debate, and resulted in the Permanent Court of International Justice being asked several questions.^39^ Paul Reinsch, arguably the most influential thinker about international organizations law,^40^ had already a decade earlier drawn attention to the difficulties involved in making labour legislation on an ad hoc basis, one treaty at the time.^41^ Hence, the organizational form was pivotal, for only a permanent organization facilitates permanent management. Only a permanent organization could manage and massage the constantly changing configurations of interests involving capital, labour and government.^42^

This dovetailed nicely with a second invention: tripartism. During World War I, the major industrialized states had all seen fit to mobilize labour and capital for the war effort, and in Britain in particular this was welcomed as an experiment well worth repeating. Britain made an effort to transplant the model to the nascent ILO, also because it realized that if it were alone among the major powers to continue to practice tripartism, it might suffer a competitive problem. Cox puts it well: “As the leading trading nation, Britain might have been disadvantaged in world markets if a peacetime prolongation of tripartism were to have the effect of raising labor costs. Hence the concern of British officials to internationalize the experiment.”^43^

One unexpected implication of the establishment of tripartism is that it cemented a place for non-state interests in the work of an international organization. It became clear that the interests of all stakeholders could not be reduced to those of the member states. This had been the traditional idea: what is good for the state, is good for everyone within the state, and things can be kept on the inter-state level. But with the ILO now, it was clearly understood that whatever the ILO would decide, adopt and promulgate, would affect workers and capital—not just the state and its competitive position. Thus, tripartism set in motion an accidental revolution, by incorporating other than direct state interests in the institutional structure of an international organization, and therewith acknowledging that the work of this organization did not just affect member states in their mutual relations, but could potentially affect every worker in every member state, and every employer in every member state.

The revolution was *accidental* in that the inspiration behind tripartism had been to secure Britain’s competitive position, rather than any grand design about popular consultation or great philosophy of the *quod omnes tangit* variety.^44^ It owed little to good intentions or to visionary inspiration. And it was a *revolution* because it opened the door to changing conceptions of international law. The establishment of the ILO slowly created the possibility for thinking of international law as directly affecting the real lives, the real interests, the real blood and real guts, of real people. If until the creation of the ILO international law could still with some sense be said to apply to inter-state relations only (if only because everyone seemed to agree that this was the case), once the ILO was created this was no longer possible: the toothpaste had been squeezed out of the tube; and once the bell tolls, its sound can no longer be unheard.

## The Changing Landscape

It is generally acknowledged that the ILO’s tripartite structure was, at the time, unique—and by and large it still is, at least in the sense in which the formal constitution of an international organization formally involves representatives of social actors other than government representatives, as the ILO does with insisting that states representations include representatives from government, labour and capital.^45^

But if the ILO’s structure is still unique, the past century has developed several variations on the same theme. In some organizations, it is possible for states to be represented by specialists: meteorologists in the case of the World Meteorological Organization; police officers in the case of Interpol (which actually started as cooperation between police forces^46^), and in the WHO there is an understanding that states strive to be represented by people with a medical background. More generally, the Universal Postal Union was the brainchild of the US Postmaster General in the 1860s, Mr. Montgomery Blair,^47^ while most of the directors-general of the International Telecommunications Union (ITU) established in 1865, have been engineers or physicists.^48^ More generally, moreover, many organizations have specialized organs where the expectation is that members have a specialist background, as with the Radio Regulations Board in ITU or the various emergency committees advising the director-general of the WHO in accordance with the 2005 International Health Regulations.^49^

In other organizations, different mechanisms are opted for. Thus, the ITU allows for corporate membership of a kind, set up much like customer loyalty schemes with several tiers; an estimated 700 companies and academic institutions thus form part of the broader ITU circle; in addition to membership by states, in this way social interests (or, by and large more accurately, corporate interests) are directly represented. The European Forest Institute has two categories of membership: state membership, and membership of research institutions (it started out as an association of research institutes), requiring an intricate institutional balance when it comes to decision-making. Some organizations participate in joint ventures with private sector actors: these are particularly prevalent in the global health domain, where an important role is played by the Bill and Melinda Gates Foundation.^50^ More generally, organizations often participate in particular projects with a range of partners from both the public and the private sectors. One prominent example is that of the Contact Group on Piracy off the Somali Coast, a somewhat loose network comprising a number of intergovernmental organizations but also comprising seafarers’ unions and, naturally perhaps, Lloyd’s of London, the leading maritime insurance company. Some organizations, moreoever, are quite dependent on financial contributions from agents other than their member states: UNHCR’s annual budget derives for some 10% from private donations, while an organization such as the International Organization for Migration (IOM) has to be largely self-sufficient, and can only do so by positioning itself as a private actor and collaborating with private actors.^51^

And then there are organizations where societal interests are represented in all sorts of advisory organs or through consultative status: think of the EU’s Committee of the Regions, or the hundreds of actors having consultative status with the UN General Assembly or the UN Economic and Social Council. Member states might be happy to include domestic actors in their national missions, whether senators or parliamentarians from the opposition or more straightforward interest representatives. And where consequential decisions are taken, lobbying is never far away. This applies not only to private interests, but also to civil society actors: it is a public secret that the Assembly of States Parties to the International Criminal Court^52^ is in thrall to the many NGOs dedicated to bringing an end to impunity, and getting various crimes and classes of victims to be recognized as relevant.

The ILO was pioneering in its tripartite structure, ensuring the representation of social interests in its standard-setting work. But it was also pioneering in a different sense: it was the first organization explicitly devoted to improving the plight of individuals, regardless of the then prevailing template according to which international organizations would only affect member state interests. At any rate, that was always an impossible conceit: it may be the case that the telegraphic pipelines regulated by the ITU were mostly publicly owned, but the senders and recipients of telegraph messages were, most often, private individuals and private companies.^53^ At the end of the day, the impact of the ITU was not just on its member states (although it was that too), but also on the citizen, the industrialist, the reporter.^54^ Likewise, the work of the UPU could not but affect those who send and receive postcards, letters and parcels from abroad—the state plays an intermediary role as a conduit for all those private interests, but it would be difficult to maintain the fiction that a missing postcard or a lost parcel would come to hurt the national interest. This was, admittedly, the prevailing mindset, but was always more ideological than real. Indeed, even the navigation rules of the early river commissions affected shipping far more than national states, and more often than not, that was the very motive behind their creation. Sayre unapologetically wrote, a century ago and at the eve of the creation of both the League of Nations and the ILO, that the various international river commissions operating in China were set up to protect western commercial interests—and these did not even bother to include China among their member states.^55^

In a sense then, by focusing on protection of workers, the ILO made explicit what was already implicit with other organizations: that the ultimate addressee and stakeholder would be the individual, whether as worker or as industrialist, with member states mostly involved as conduit. The member states make the rules together and have to implement them in one way or another, but it would be insufficient to say that the regime only affected those member states, and not any one residing within them. With the ILO this was, no doubt, the result of turning vice into virtue: the focus on the individual was occasioned by the distrust of other states. The risk of facilitating “free riding” was simply too big to organize worker protection in any other way than through the combination of continuous law-making while respecting sovereignty, and to do so through a permanent entity rather through a single convention or small group of related conventions.

It was only once the ILO had sensitized international law to the possibility of piercing through the mystifying veil of the state, that the international community could come to think of protecting human rights. And even then it took a while still, with direct protection of individual human rights hesitantly^56^ emerging in the late 1940s and early 1950s and, importantly, after another World War had underlined that perhaps concerted action would be required to prevent further atrocities, and states could not be relied on to do so themselves.^57^ The Universal Declaration, the European Convention on Human Rights, the Genocide Convention and the Refugee Convention, they were all concluded within a period of 3 years or so (1948–1951), and all have protection of the individual as their common topic. Importantly though, they all envisage a conduit role for the state, and to the extent that international monitoring was put in place, it would be considerably later, and typically on a voluntary basis, through additional optional protocols. The point for present purposes though is that these instruments were only possible once the ILO had opened the windows and let in a fresh breeze, diluting the stale air of a strong inter-state conception of international law.

This would be further developed by the EU, that wonderful and occasionally somewhat tragic experiment in governance and authority beyond the state.^58^ The original treaties, concluded in the 1950s, already manifested that public and private participation were envisaged, for instance in the form of the revolutionary creation of the European Parliament. But the EU went considerably further, as its Court of Justice (itself open to other than inter-state complaints) acknowledged in a string of classic cases, including *Van Gend & Loos* and *Costa* v *ENEL*, both decided early in the EU’s existence.^59^ The existence of a preliminary reference procedure, allowing domestic courts to consult the CJEU, was pivotal, as was the positing of the direct effect of EU law in the domestic legal orders of the member states. The legal instruments envisaged would create Union-wide legislation (or at least harmonize the domestic laws of the member states), and the Commission would have enforcement powers across national boundaries. The EU truly marked an astonishing experiment, but it is important to note that, as with most other experiments, it stands on the shoulders of predecessors: the EU would have looked different, and possibly less adventurous, without the earlier pioneering work that went into creating the ILO.

This is so not only because its main *auctor intellectualis*, Jean Monnet, worked for a while close by the ILO as the Deputy Decretary-General of the League of Nations, and may have had a look at how the ILO was set up and how it worked in practice.^60^ It is also not only because the EU was created with considerable Christian-democrat input, and had an ideological affinity for social cooperation between stakeholders in accordance with Christian doctrine.^61^ This may have been given an extreme form earlier by Mussolini, turning corporatism into fascism, but the basic corporatist idea so central to Christian political philosophy characterizes both the ILO and the EU—albeit probably for different reasons.^62^

But the main reason why the EU would have looked differently without the ILO experience is the circumstance highlighted above: the ILO was the first to clear the state-centric cobwebs from international organization, and the first to open up international law to recognition and embrace of interests other than those presumed to be of states. One might argue, of course, that states have few interests of their own, other than the circular concept of the *raison d’état*. That is an insight that is slowly gaining acceptance, but credit where credit is due: possibly the first venue where this was made visible was the ILO, partly because it incorporated social interests through its tripartite structure, and partly because it may well have been the first venue (forced by circumstances, but nonetheless…) which recognized that individuals could be addressed under international law. While there is some ground to suggest that the bilateral treaties of the nineteenth century aided in bringing slavery to an end, these treaties were still the result of paternalist impulses. What made the ILO different was that to the extent that paternalist thought was involved, it was counterbalanced by the self-interests of employers. The ILO took the form, eventually, of states making law to protect individuals, but beneath this surface layer, the ILO’s output is the outcome of serious social struggle between labour and capital—governance beyond the state, rather than governance between states.

## To Conclude

It may well be the case that, as far as the concrete standard-setting and effectiveness thereof is concerned, the ILO may have become somewhat marginalized over the course of its first century.^63^ There are some topics related to labour where one wishes it would have been a little more active, a little more vocal—the link between labor and migration comes to mind, which can scarcely be left to individual governments alone or to the International Organization for Migration, with its mandate to ensure orderly migration but with less of a humanitarian impulse governing its activities. Likewise, the ILO may still be adapting to transformations of the global political economy, with global supply chains and the emergence of the platform economy changing the scene.^64^

But even so, the world would look differently, and most likely considerably worse, without the ILO. Its main contribution has not just been in concrete standard-setting, but perhaps even more so, as this paper has argued, in opening up the closed universe of inter-state international law, therewith paving the way for later developments,^65^ including refugee protection, human rights protection, and the emergence of the EU. And that is, by any standard, quite an achievement.

## Footnotes

1 Klabbers (2015a), pp. 488–506.

2 See European Court of Human Rights (somewhat softened by the Grand Chamber), *Perinçek v. Switzerland* (App. No. 27510/08), Judgment, 15 October 2015; for discussion, see Klabbers (2017a).

3 This refers to the classic study by Kratochwil (1989). See also Kratochwil (2018).

4 Klabbers (2020).

5 Lukes (1974).

6 Keynes (1920), p. 279: “[…] the true voice of the new generation has not yet spoken, and silent opinion is not yet formed. To the formation of the general opinion of the future I dedicate this book.”

7 I will studiously refrain from using the term ‘subject’ of international law, as this often merely functions as a placeholder.

8 Oppenheim (1912), p. 3. Oppenheim’s influence can hardly be over-estimated: see García-Salmones Rovira (2013).

9 Oppenheim (1912), p. 516.

10 Think of the Holy See or the Maltese Knights. Intriguingly, upon its creation the League of Nations was categorized in the same manner in one of the great historical overviews of international law. See Verzijl (1969).

11 Klabbers (2015b).

12 Klabbers (2016), pp. 618–634.

13 International Court of Justice, *Reparation for Injuries Suffered in the Service of the United Nations*, Advisory Opinion, 11 April 1949. In International Court of Justice Reports of Judgments, Advisory Opinions and Orders 1949. See also Klabbers (2017b), pp. 105–121.

14 Permanent Court of International Justice, *Case of the Mavrommatis Palestine Concessions*, Judgment, 30 August 1924. In Publications of the Permanent Court of International Justice, Series A, No. 2.

15 Jessup (1948), p. 9.

16 And when the UK started to pioneer the prosecution of slave traders, it did so largely on the basis of its domestic law, even if to some extent bilateral treaties proved supportive. See Martinez (2014).

17 Note how, as so often in legal thought, the conclusion is already inherent in the premises.

18 Triepel (1899).

19 Klabbers (forthcoming).

20 Triepel (1923), pp. 73–121.

21 Bennett (2005).

22 The latter is memorably depicted in Koskenniemi (2001).

23 It eventually joined in 1908.

24 Jessup (1956), p. 17.

25 See also the illuminating study by Fakhri (2014).

26 Borchard (1940), p. 139.

27 Permanent Court of International Justice, *Jurisdiction of the Courts of Danzig*, Advisory Opinion, 3 March 1928. In Publications of the Permanent Court of International Justice, Series B, No. 15.

28 And this in turn has been put to effective use by some states in the form of so-called “non-self-executing declarations”. See further Klabbers (2017c), pp. 325–326.

29 Note however that the League too could not avoid addressing the plight of individuals, in particular those living under the Mandate system. For a fine historical analysis, see Pedersen (2015).

30 Morse (1969), p. 4. Contrast this with another view: “The spectre of Bolshevism was a powerful stimulus for being responsive to the requests of labor.” Jacobson (1984), p. 302.

31 Note that some of these (night work, phosphorus) had already been the subject of conventions concluded during the early 1900s under auspices of the International Association for Labour Legislation.

32 Barnes (1926), p. 45.

33 Ibid., p. 37.

34 Ibid., p. 45. By Eastern, he meant Asian.

35 Woolf (1916), p. 183.

36 Hobson (1915), p. 143.

37 Klabbers (2019a), pp. 629–646.

38 Rosenberg (2012), p. 35. Mazower agrees, noting that the ILO followed “a precarious corporatist course between hostile capitalists to its right and revolutionary socialists to its left”. Mazower (2012), p. 152.

39 Permanent Court of International Justice, *Competence of the ILO in regard to International Regulation of the Conditions of Labour of Persons Employed in Agriculture*, Advisory Opinion, 12 August 1922. In Publications of the Permanent Court of International Justice, Series B, No. 2; Permanent Court of International Justice, *Competence of the ILO to Examine Proposal for the Organization and Development of the Methods of Agricultural Development*, Advisory Opinion, 12 August 1922. In Publications of the Permanent Court of International Justice, Series B, No. 3. It also took a few years for the Court itself to come to terms with the institutional element: it only started to develop a theory of powers in Permanent Court of International Justice, *Competence of the ILO to Regulate Incidentally the Personal Work of the Employer*, Advisory Opinion, 23 July 1926. In Publications of the Permanent Court of International Justice, Series B, No. 13.

40 On the relevance of Reinsch, see Klabbers (2014a).

41 Reinsch had noted that the International Association of Labor Legislation was “admirably fitted” for harmonizing and unifying labour legislation, probably precisely because of its permanence—although he shied away from drawing the conclusion explicitly. Reinsch (1911), p. 47.

42 Maupain hints at much the same when discussing the difficulties inherent in making international labour legislation, resulting either in free riding (and thus disturbing competitive balances) or in the adoption of the lowest common denominator. As a result, what was needed was a constitutional structure that allowed for persuasion while leaving sovereign prerogatives intact. See Maupain (2013), p. 15.

43 Cox (1987), p. 75.

44 This is the sort of trope (*quod omnes tangit ab omnibus approbatur*; “what touches all should be approved by all”) that might come to play a role in different settings. One well-known manifestion is the slogan “no taxation without representation”.

45 This, in turn, may give rise to domestic struggles about who gets to represent labour and capital; those struggles sometimes reach international tribunals, and have done so right from the start: Permanent Court of International Justice, *Designation of the Workers’ Delegate for the Netherlands at the Third Session of the International Labour Conference*, Advisory Opinion, 23 July 1926. In Publications of the Permanent Court of International Justice, Series B, No. 1.

46 Martha (2010).

47 Sly (1927), pp. 395–436.

48 Klabbers (unpublished paper, 2019, on file with the author).

49 On the latter, see Klabbers (2019b).

50 Andonova (2017).

51 Klabbers (2019c).

52 Note that for these purposes there is no problem in treating the ICC and its ASP as an international organization. For other purposes doing so might be less easily justifiable: to the extent that organizations exercised delegated powers and act under instructions from their member states, one might be reluctant to include judicial institutions.

53 In his pathbreaking study, Murphy demonstrates just how strongly the ITU has been the pivot around which the first global telecommunications revolution revolved in the late nineteenth century, effectively establishing the global legal infrastructure for the entire business. See Murphy (1994).

54 It is hardly a coincidence that around the same time, international journalism came off the ground, and Reuter’s started to become a household name. See, e.g., Wilson (2016).

55 Sayre (1919).

56 Some suggest it was not until the 1970s that human rights seriously became successful. See Moyn (2010).

57 The interbellum minorities treaties were always exceptional: the ambition was not so to protect individuals, but to counterbalance the viccisitudes of great power politics at Versailles.

58 Klabbers (2019d), pp. 25–41.

59 European Court of Justice, *NV Algemene Transport- en Expeditie Onderneming van Gend & Loos v. Netherlands Inland Revenue Administration* (Case 26/62), Judgment, 5 February 1963; European Court of Justice, *Flaminio Costa* v *E.N.E.L* (Case 6-64), Judgment, 15 July 1964.

60 Monnet’s biographer does not discuss the ILO, but does cite Monnet’s confession that while at the League, he “did not understand the politics of Versailles, only the economics”. Duchêne (1994), p. 364.

61 While not stressing the Christian-democrat element, an excellent discussion of the ordo-liberalism that went into the EU and later the WTO is Slobodian (2018).

62 See Cox (1987), p. 101. Above I explain that tripartism (the ILO’s version) owes much to British interests; that logic cannot apply to the EU, which saw the light without much British participation.

63 Klabbers (2014b), pp. 181–196.

64 See Berliner et al. (2015) and Srnicek (2016).

65 A different question is what sort of individual is sponsored and supported by international law: see Urueña (2012).
